# Protocol for population testing of an Internet-based Personalised Decision Support system for colorectal cancer screening

**DOI:** 10.1186/1472-6947-10-50

**Published:** 2010-09-16

**Authors:** Carlene J Wilson, Ingrid HK Flight, Ian T Zajac, Deborah Turnbull, Graeme P Young, Stephen R Cole, Tess Gregory

**Affiliations:** 1 Flinders Centre for Cancer Prevention and Control, School of Medicine, Flinders University, Sturt Road, Bedford Park, SA 5042, Australia; 2 Cancer Council South Australia, 202 Greenhill Road, Eastwood, SA 5063, Australia; 3 Colorectal Cancer and Gut Health, Preventative Health Research Flagship, Commonwealth Scientific and Industrial Research Organisation, Gate 13, Kintore Avenue, Adelaide, SA 5000, Australia; 4 School of Psychology, University of Adelaide, North Terrace, SA 5000, Australia; 5 Bowel Health Service, Repatriation General Hospital, Daws Road, Daw Park, SA 5042, Australia

## Abstract

**Background:**

Australia has a comparatively high incidence of colorectal (bowel) cancer; however, population screening uptake using faecal occult blood test (FOBT) remains low. This study will determine the impact on screening participation of a novel, Internet-based Personalised Decision Support (PDS) package. The PDS is designed to measure attitudes and cognitive concerns and provide people with individually tailored information, in real time, that will assist them with making a decision to screen. The hypothesis is that exposure to (tailored) PDS will result in greater participation in screening than participation following exposure to non-tailored PDS or resulting from the current non-tailored, paper-based approach.

**Methods/design:**

A randomised parallel trial comprising three arms will be conducted. Men and women aged 50-74 years (*N *= 3240) will be recruited. They must have access to the Internet; have not had an FOBT within the previous 12 months, or sigmoidoscopy or colonoscopy within the previous 5 years; have had no clinical diagnosis of bowel cancer. Groups 1 and 2 (PDS arms) will access a website and complete a baseline survey measuring decision-to-screen stage, attitudes and cognitive concerns and will receive immediate feedback; Group 1 will receive information 'tailored' to their responses in the baseline survey and group 2 will received 'non-tailored' bowel cancer information. Respondents in both groups will subsequently receive an FOBT kit. Group 3 (usual practice arm) will complete a paper-based version of the baseline survey and respondents will subsequently receive 'non-tailored' paper-based bowel cancer information with accompanying FOBT kit. Following despatch of FOBTs, all respondents will be requested to complete an endpoint survey. Main outcome measures are (1) completion of FOBT and (2) change in decision-to-screen stage. Secondary outcomes include satisfaction with decision and change in attitudinal scores from baseline to endpoint. Analyses will be performed using Chi-square tests, analysis of variance and log binomial generalized linear models as appropriate.

**Discussion:**

It is necessary to restrict participants to Internet users to provide an appropriately controlled evaluation of PDS. Once efficacy of the approach has been established, it will be important to evaluate effectiveness in the wider at-risk population, and to identify barriers to its implementation in those settings.

**Trial registration:**

Australian New Zealand Clinical Trials Registry ACTRN12610000095066

## Background

In Australia, the risk of being diagnosed with colorectal cancer (CRC) by the age of 85 years is 1 in 10 for males and 1 in 14 for females, with the risk increasing sharply from the age of 45 years [1]. In 2006, CRC accounted for 10% of all invasive cancer-related deaths, second only to lung cancer [2]. Despite evidence from randomised clinical trials that both biennial and annual screening using faecal occult blood testing reduces CRC mortality [3-5] and incidence [6], the participation rate remains low. Since 2007 the Australian government-funded National Bowel Cancer Screening Program (NBCSP) has been operational and now provides people turning 50, 55 and 65 years with a free Faecal Occult Blood Test (FOBT) kit. This home-based test involves sending a stool sample to a laboratory to be analysed for occult blood, ideally followed by colonoscopy for those with a positive result. The 2008 participation rate was 39% of the eligible population [1], with uptake varying between sexes (42% for females compared to 36% for males). These suboptimal rates highlight the public health challenge to optimise participation rates using publicly delivered rather than clinician-based interventions.

An understanding of the variables that encourage people to participate in CRC screening using FOBT is important for a number of reasons. Participation rate is the critical determinant of population efficacy of screening as it improves outcomes independent of screening technology. Early detection and treatment of precancerous lesions and adenomas results in a significantly higher survival rate than if treatment is delayed until physical symptoms of the conditions are evident [4]. Moreover, the cost effectiveness of FOBT screening is expected to increase as greater participation and earlier detection of CRC reduces treatment costs and improves longer-term survival rates [7].

Poor participation in screening can be attributed to several factors including a poor understanding of cancer and cancer risk [8], insufficient knowledge of CRC and the value of screening [9,10] and a perception of the screening process as distasteful or embarrassing [11,12]. Decision aids have been developed to overcome some of the problems associated with poor participation in cancer prevention programs including participation in CRC screening. Their purpose is to assist the decision making of people facing health treatments or screening decisions through increasing knowledge of and awareness about personal risk and response alternatives. Screening aids have been shown to improve knowledge, reduce decisional conflict and stimulate individuals to be more active in decision making without increasing their anxiety [13,14]. Furthermore, a meta-analysis of the effectiveness of these aids confirms their utility for improving knowledge of cancer screening by comparison to usual practice [15]. The utility of these aids is likely to be affected by several factors including the theoretical framework underpinning the aid, the format of the decision aid and the nature and presentation of the information.

### Theoretical framework

Two classes of behavioural theory hold particular relevance to the development of decision aids designed to enhance screening participation. These are described as "continuum" and "stage" theories [16]. Continuum theories focus on the exploration of psychosocial predictors of intention to screen; a recent variant, the Preventive Health Model (PHM), draws from other theoretical models including the Health Belief Model [17]. The PHM indicates that individual differences in preparedness to screen for CRC can be attributed to scores on five factors: salience and coherence (the extent to which performing CRC screening is consistent with beliefs about how to protect and maintain health); cancer worries (concerns about the consequences of CRC); response efficacy (beliefs that undertaking CRC screening will be effective in reducing disease threat); social influence (beliefs about, and desire to comply with the attitudes of key others to CRC screening); and perceived susceptibility (subjective personal risk for developing CRC). These constructs have been validated in the U.S. [18,19] and Australia [20]. It has been argued that the PHM is enhanced when utilised with a "stage" theory such as the Precaution Adoption Process Model (PAPM) [21] which deconstructs behavioural intention and posits that individuals are more likely to respond to interventions aimed at their stage of readiness to engage in screening. The PAPM describes people as being at one of seven stages: unaware of the issue, heard of the issue but unconcerned, considering action, decided against a behaviour, decided to act, acting and lastly, maintaining the behaviour over time. Myers and colleagues have demonstrated that groups of people at a specific stage of thinking about colorectal cancer screening can be distinguished from people at a higher level in terms of their responses to the variables included in the PHM [22]. For example, for those people who have "never heard of" FOBT (unaware of the issue), messages which emphasise the PHM factors of salience and coherence and perceived susceptibility are more likely to move them toward a decision to screen, whereas messages focussing on perceived susceptibility and self efficacy are more likely to resonate with those who have "decided against" screening and encourage them toward screening.

### CRC screening decision aid format and the presentation of information

The PAPM and PHM models provide the basis upon which to develop an understanding of the cognitive frame of reference that underlies an individual's decision-making around CRC screening. Research suggests that the effectiveness of decision aids is enhanced when the information provided is tailored to the needs of the individual; the communications are consistently better remembered and perceived as being more relevant than non-tailored materials [23]. Tailoring messages according to current decision stage (PAPM), and current responses on the full range of psychosocial drivers measured in models like the PHM, should enhance participation, maximise a person's satisfaction with their decision, and minimise any feelings of anxiety or dissonance ("decisional conflict").

Paper-based delivery of tailored messages has improved screening uptake [24] but the feasibility of this approach, if implemented on a large scale, has yet to be established. A meta-analysis comparing web-based and non-web-based informational interventions showed enhanced outcomes among individuals using web-based interventions, particularly in the areas of knowledge and targeted behaviour change [25]. The potential advantages of web-based delivery over traditional paper methods include the ability to present information to the reader in a way that is more easily navigable than on paper; the capacity to use context to enhance relevance through the process of tailoring on key variables; and the ability to provide instantaneous enactment of the decision reached, thereby avoiding the difficulties that are attached to procrastination (for example, screening tests could be ordered instantly online or an appointment made with a doctor via email). Thus, there is a need to test whether electronic delivery can achieve similar or improved uptake rates compared to paper-based delivery, particularly within a community setting.

A functional Internet-based Personalised Decision Support system (PDS) developed by the Commonwealth Scientific and Industrial Research Organisation (CSIRO) Preventative Health Flagship is available for this study. It is an interactive application that collects user information in real-time and delivers instantaneous, personalised messages aimed at moving individuals through the decision stages relevant to CRC screening. An extensive User Model drives a series of algorithms that underlie an educational message library. These algorithms have been developed to ensure that messages delivered to an individual are united with natural language so that they can be read in a coherent, logical manner. After log-in, individuals complete a questionnaire that incorporates PAPM and PHM variables. Items are rated on a 5-point Likert scale from 'strongly disagree' to 'strongly agree'. The computer program processes these data to determine the individualised content to be delivered following completion of the questionnaire. The resulting web interface consists of tailored, personalised messages that address relevant knowledge deficits and reinforce perceptions when favourable to screening, or motivate people to change perceptions when unfavourable. For example, a library of messages has been created to address response efficacy, a factor which relates strongly to those who are "not considering" FOBT screening. The response efficacy statement reads: "When colorectal polyps are found and removed, colorectal cancer can be prevented". Participants who respond "disagree" receive a personalised 'motivating' message reading; "[Participant's name], *you don't believe that colon cancer screening is effective. In fact, it's very effective ...*" Participants who respond '"agree" would receive a 'reinforcing' message: "*You've told us that you believe colon cancer screening is effective. You're right ...*" Messages are also presented in a specific order so that messages relating to PHM factors which are most strongly associated with an individual's current decision stage [22] are presented first with the aim of moving people through stages of screening awareness and pre-screening contemplation to a decision to participate.

In conclusion, communicating the necessity for CRC screening and the effectiveness of screening tests available to the target population is a clearly demonstrated need. Tailored decision support, delivered via the Internet, holds the prospect of improving participation rates and thereby health outcomes into the future.

## Methods/Design

### Study Aims

The primary aims of the study are to test whether (1) an Internet-based, Personalised Decision Support package (PDS) that delivers personalised information, tailored according to preventive health variables and current decision stage for screening enhances FOBT participation when compared to a non-tailored PDS package and the current paper-based approach; and (2) whether tailored PDS moves individuals to a higher decision stage for screening compared with the other interventions.

### Study design and setting

**Figure 1:**
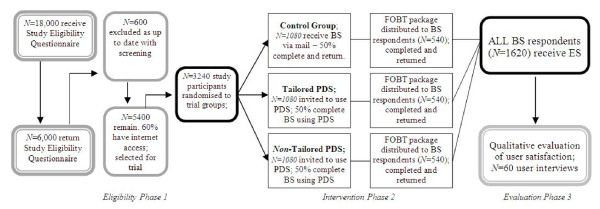
**Trial design flow chart and estimated attrition rates**.

This study is a parallel group, randomised trial where a randomly-selected, national sample of 3240 men and women aged 50 to 74 years who are known to have Internet access are randomised to a tailored or non-tailored PDS, or a non-tailored, paper-based approach (usual practice). Only one member from each household will be recruited to avoid cross-contamination of the groups. The trial design flowchart, with estimated attrition rates, is shown in Figure 1.

#### Ethical considerations

Full ethical approval has been obtained from the CSIRO Human Research Ethics Committee.

### Identification of eligible participants

We will approach a randomly selected, national mainland Australian sample of *N *= 18,000 urban dwelling, men and women aged 50-74 years identified from the electoral roll. The sample will be stratified according to population density at the Australian State level. Rural areas and Tasmania will not be included because the former will have added constraints associated with potential difficulties in accessing gastroenterological services and the latter has a population too small to allow for appropriate stratification. Potential participants will be approached via a posted Eligibility Questionnaire (EQ) and information sheet outlining the nature and requirements of the study and asking them to address the inclusion and exclusion criteria if they are interested in participating. A pre-paid return envelope will be enclosed for return of the documents. There will be an opportunity for people to opt out at this stage.

#### Determining eligibility for the study

Potential participants will be selected from those who return the EQ and who are eligible based on criteria shown in Additional File 1.

#### Randomisation

Those who have indicated, by return of the completed EQ, that they have read the study information, are eligible and willing to participate in the study will be randomly assigned to one of three arms (Figure 1). Block randomisation using random block size will be utilised, and stratification will be performed to ensure that the numbers of participants receiving each intervention are closely balanced within each Australian State.

#### Blinding

Data collectors for the primary outcome (FOBT uptake) will be blinded to treatment arm allocation following randomisation. Data analysts will be blinded to treatment arm allocation and will become unblinded following analysis of the primary outcomes. Participants and investigators will not be blinded.

### Study interventions

The study involves three arms: (1) CRC information and invitation to screen via Internet access to 'tailored' PDS; (2) CRC information and invitation to screen via Internet access to 'non-tailored' PDS; and (3) paper-based delivery of CRC information and invitation to screen, consistent with usual practice in the National Bowel Cancer Screening Program (NBCSP). Interventions specific to each arm and study stage are shown in Table 1 and described below. All groups will receive a second copy of the information sheet and have the opportunity again to opt out at this stage.

**Table 1:** Study interventions by phase and arm

Eligibility Phase 1	Arm	Intervention Phase 2.1	Intervention Phase 2.2	Evaluation Phase 3.1	Evaluation Phase 3.2
Information Sheet + Eligibility Questionnaire (EQ) then eligible participants randomised to study Arm	Internet-based Tailored PDS	Information Sheet Baseline survey (BS) Receipt of tailored messages Electronic version of NBCSP consumer information booklet	FOBT kit received Reminder to revisit tailored messages and consumer information booklet	Endpoint survey (ES)	Telephone qualitative interview (subset)
	
	Internet-based non-tailored PDS	Information Sheet Baseline Survey Electronic version of NBCSP consumer information booklet	FOBT kit received Reminder to revisit consumer information booklet	Endpoint survey	Telephone qualitative interview (subset)
	
	Paper-based non-tailored (usual practice)	Information Sheet Baseline Survey	FOBT kit received Printed version of NBCSP consumer information booklet	Endpoint survey	Telephone qualitative interview (subset)

Participants in each of the two PDS arms will be required to access the PDS web site and log in with a unique user name and ID provided by the researchers. Once logged on, participants in both arms will complete a Baseline Survey (BS) that will collect demographic data, PAPM decision stage and measure PHM variables. Participants in the *Tailored PDS *arm will receive appropriately motivating or reinforcing messages tailored to their responses to the BS variables. In addition they will be able to access an electronic version of the NBCSP's consumer information booklet [26]. This provides information about the NBCSP, including information on bowel cancer, screening, and completing the FOBT kit. In the *non-tailored PDS *arm, participants will not receive any tailored messages upon completion of the BS but they will be able to access the NBCSP consumer information booklet. In the *paper-based *usual practice arm, the BS only (without an accompanying consumer information booklet) will be offered by mail. At that stage, all participants will be informed that they will receive an FOBT kit approximately 2 weeks after completion of the BS. This information will equate to the 'pre-notification' process employed in the NBCSP.

All participants who have not opted out of the trial at the BS phase and have completed a BS will receive a commonly used FOBT kit of the immunochemical type (Faecal Immunochemical Test, FIT) that does not require dietary or drug restrictions, and an invitation to screen. In the usual practice arm, the offer will consist of a mailed, personalised (by name and address) invitation to screen and will include printed educational material in the form of the NBCSP consumer information booklet. The offer to PDS participants will parallel that of the usual practice arm, with the distinction that the printed educational material will not be provided but both groups will be reminded that they may return to the PDS using their supplied username and password to re-visit messages (tailored arm) and the electronic version of the NBCSP consumer information booklet. A reminder will be sent 6 weeks after despatch of FOBT to those who have not returned their kit.

Participants will be contacted by letter 1-2 weeks after receipt of their completed FOBT, or 12 weeks following the invitation to screen for those who haven't returned their kit during this period, and requested to complete an Endpoint Survey (ES) either by returning to the online site (PDS arms) or completing the accompanying ES in paper format (usual practice arm). The ES will re-measure the PHM/PAPM variables included in the BS and collect additional information relating to the outcome measures (Table 2).

**Table 2:** Data collection stages

**Phase 1 Eligibility Questionnaire, self-completed on paper. Reminder sent 2 weeks after initial mailing**.
Questions addressing the inclusion and exclusion criteria (Additional file 1)
**Phase 2.1 Baseline Survey, self-completed on line or on paper as allocated. Reminder sent 2 weeks after initial mailing**.
Demographic characteristics
Age, sex, education, country of birth, marital status, employment status
PAPM stage
Decision stage for screening assessed by PAPM stage (never heard of FOBT, aware but not engaged, decided not to act, undecided, decided to act)
PHM constructs
Scores assessed on salience and coherence, cancer worries, response efficacy, social influence, perceived susceptibility [18]
Self efficacy
Score assessed on confidence to use an FOBT at home
Faecal aversion
Score assessed on distaste or embarrassment toward handling of faeces

**Phase 2.2 Invitation to screen, including FOBT. Sent 2-4 weeks after completion of baseline survey with reminder sent 6 weeks after initial invitation**.
Receipt or non-receipt of completed FOBT recorded by the Bowel Health Service (BHS), Repatriation General Hospital
Return of kit
Date of return of kit
Number of participants who contact the 'help line' provided as part of the standard BHS protocol

**Phase 3.1 Endpoint Survey, self-completed on line or on paper as allocated. Administered 1-2 weeks following receipt of completed FOBT, or 12 weeks following invitation to screen. Reminder sent 2 weeks after initial mailing**.
PAPM stage
PHM construct scores
Self efficacy score
Faecal aversion score
Participation in any other bowel cancer screening activity since entry into trial
Familial history of bowel cancer
Usefulness of the educational material
Satisfaction with content and ease of navigation (PDS arms)
Decisional satisfaction and conflict. Scores assessed by the Decisional Conflict scale [32]
Level of motivation to screen. Scores assessed by the Treatment Self-Regulation Questionnaire [33]

**Phase 3.2 Qualitative data, obtained following Endpoint Survey from telephone interviews with a subset of participants in each arm**.
Reasons for choosing to participate or not in screening
Usefulness of materials
How the interventions might be improved

Qualitative research will be undertaken with a subset of participants from each arm who will be asked to participate in a one-to-one telephone interview. Participants will be selected with a wide variation in demographic, psychological and behavioural characteristics (ie we will seek to interview screening responders and non-responders). The semi-structured, open-ended interviews will explore reasons for invitees choosing to engage, or not, in CRC screening.

### Participant follow-up procedures

The study consists of five stages of data collection (Table 2). All participants will be followed up from study entry until receipt of the ES, with a sub-set of those who have completed and not completed an FOBT being invited to participate in a telephone interview following the ES.

#### Participant outcome measures

The primary outcomes are (1) return of completed FOBT within 6 weeks (prior to reminder) and 12 weeks (after reminder); and (2) change in decision stage on the PAPM between intention expressed in the BS and decision stage as measured in the ES. Key secondary outcomes are (1) change in participant responses to the PHM variables between completion of the BS and the ES; (2) decisional conflict and satisfaction scores; and (3) satisfaction with the screening process. These and other measures are summarised by collection stage in Table 2.

### Sample size considerations

Randomisation of 3240 participants (1080 in each arm) takes into consideration predicted attrition of 50% at BS survey phase, followed by screening offers being distributed to 1620 survey respondents (540 in each arm). This permits detection of differential FOBT uptake of at least 10% between two groups (e.g., 40% vs 50%) with 80% power and alpha of 0.05. To recruit this number of participants, an initial sample of 18,000 needs to be approached, given our estimated attrition rates throughout the study (Figure 1).

### Statistical analysis

#### Primary outcomes

FOBT participation will be compared between arms using a Chi-squared test. The change in coefficient method using log binomial generalized linear models (GLM) will then be used to check for potential covariates (for example, PHM factors, decisional conflict, gender, SES, age band, Internet access characteristics and so on) and to provide a final adjusted comparison of screening uptake rates. Change in decision stage will be analysed using analysis of covariance based on PAPM scores to compare the decision stage after the intervention having adjusted for the baseline stage.

#### Secondary outcomes

Changes in participant responses to the PHM variables will be examined for their relationship to change in PAPM decision stage and FOBT participation. Log binomial GLMs will be used to determine the best joint predictors of participation. Scores on decisional conflict and satisfaction variables will be compared between the arms using a chi-squared test or Students t-test as appropriate. User satisfaction with the screening process will be assessed by qualitative research; the Framework analysis approach [27] will be utilised as it is suited to applied research that incorporates *a priori *issues and that intends to make recommendations for practice. Content analysis [28] will also be used to identify key themes and concepts.

### Time plan for study

Participant recruitment began in March 2010 and is planned to continue to July 2010. The final stage of the study, the qualitative evaluation, is planned to be complete by June 2011. As at September 1^st^, *N *= 3240 (100% of target) had been recruited into the study.

## Discussion

The study aims to test the *efficacy *of the intervention in participants where the intervention and the screening process seem feasible. We acknowledge that requiring participants to have access to the Internet may raise concerns about health equity. However, it is necessary to restrict participants to Internet users to provide an appropriately controlled evaluation of PDS. Moreover, we do not consider this restriction will represent an undesirable degree of selection bias as the results of an earlier study we conducted found that more than half the population over 50 years in South Australia has access to the Internet at some location [29]. Likewise, participation is limited to those living in urban postcode regions. This is because those who have a positive FOBT result will be more likely to achieve speedy access to gastroenterological services [30] and incur fewer personal costs and difficulties [31]. Restriction of study participants to those living in urban areas will avoid confounding of the results by service accessibility issues. Once efficacy of the approach has been established, it will be important to evaluate effectiveness in the wider at-risk population, including rural regions, and to identify barriers to its implementation in those settings.

## Competing interests

The authors declare that they have no competing interests.

## Authors' contributions

CW, IF, DT, GY and SC were responsible for identifying the research question, and contributing toward the drafting of the study protocol. CW and IF were responsible for developing and piloting the initial decision support tool and evaluating usability and acceptability. IZ and TG contributed to the development of the protocol and study design utilised in the funding application, and IZ has been appointed as Trial Manager for this study. IF was primarily responsible for the drafting of this manuscript with all authors providing comments on drafts and approving the final version.

## Pre-publication history

The pre-publication history for this paper can be accessed here:

http://www.biomedcentral.com/1472-6947/10/50/prepub

## Supplementary Material

Additional file 1 **Study inclusion and exclusion criteria**. Details of participant eligibility criteria Click here for file
