# Supplementary material for: Protocol for population testing of an Internet-based Personalised Decision Support system for colorectal cancer screening
Source: BMC Med Inform Decis Mak. 2010 Sep 16;10:50. doi: 10.1186/1472-6947-10-50 (PMC2949693; doi:10.1186/1472-6947-10-50)
Supplement: Additional file 1 — Study inclusion and exclusion criteria. Details of participant eligibility criteria [file 1472-6947-10-50-S1.DOC]

Additional File 1. Study inclusion and exclusion criteria

| *Inclusion criteria* |
| --- |
| Aged between 50 and 74 years.  Access to the internet at some location (for example, at work, at home, at a family member’s house, library). |
| *Exclusion criteria* |
| FOBT screening within the previous 12 months.  Sigmoidoscopy or colonoscopy within the previous 5 years.  Clinical diagnosis of bowel cancer. |
